# Anticoagulant therapy is not a risk factor for choroidal haemorrhage

**DOI:** 10.1007/s00417-014-2807-z

**Published:** 2014-10-03

**Authors:** Andrzej Grzybowski, Somdutt Prasad

**Affiliations:** 1 Department of Ophthalmology, Poznan City Hospital, ul. Szwajcarska 3, 61-285 Poznań, Poland; 2 Department of Ophthalmology, University of Warmia and Mazury, ul. Żołnierska 14C, Olsztyn, Poland; 3 B B Eye Foundatin, 2/5, Sukhsagar, Sarat Bose Road, Kolkata, 700020 India; 4 Rotary Narayana Nethralaya, Salt Lake, Sector V, Kolkata, 700091 India

Dear Editor,

Jin et al. report the experience of the management of delayed supra-choroidal haemorrhage (DSCH) [1]. Table 1 in their paper lays out the risk factors, procedures, and outcomes, but does not mention usage of anticoagulation and antiplatelet aggregation agents. Based on one patient who is stated to have been on Warfarin, they go on to conclude that Warfarin usage is a major risk factor for DSCH, and also recommend cessation of warfarin for 4 days to allow resynthesis of coagulation factors, and cessation of other antiplatelet agents (such as clopidogrel and ticlopidine) for 2 weeks.

We think it is important to point out that clear corneal phacoemulsification under topical anaesthesia can be safely done without stopping warfarin or antiplatelet agents [2, 3]. The American Academy of Ophthalmology’s Preferred Practice Pattern on this subject also states that anticoagulation with warfarin does not significantly increase the risk of choroidal haemorrhage [4,] as was also shown by the British National Cataract Dataset report [5]; and the UK Royal College of Ophthalmologists also does not recommend stopping anticoagulation or antiplatelet agents for cataract surgery, as doing so may increase the risk of stroke and death. [6]

We would therefore like to emphasise that stopping anticoagulation or antiplatelet agents for routine phacoemulsification is against available evidence and guidelines, and therefore should not be done, although it is sensible to check that the INR is in the desired therapeutic range as set by the treating physician, and the surgery should be done under topical anaesthesia by an ophthalmologist used to doing the operation with this technique; if need be, sub-Tenon anaesthesia may be used.
